# Sulfur Mustard Research—Strategies for the Development of Improved Medical Therapy

**Published:** 2008-06-10

**Authors:** Kai Kehe, Frank Balszuweit, Judith Emmler, Helmut Kreppel, Marianne Jochum, Horst Thiermann

**Affiliations:** Bundeswehr Institute of Pharmacology and Toxicology, Neuherbergstr. 11, 80937 Munich, Germany; Division of Clinical Chemistry and Clinical Biochemistry, Surgical Department, City of the Ludwig-Maximilians-University, Nussbaumstr 20, D-80336 Munich, Germany.

## Abstract

**Objective:** Sulfur mustard (SM) is a bifunctional alkylating substance being used as chemical warfare agent (vesicant). It is still regarded as a significant threat in chemical warfare and terrorism. Exposure to SM produces cutaneous blisters, respiratory and gastrointestinal tract injury, eye lesions, and bone marrow depression. Victims of World War I as well as those of the Iran-Iraq war have suffered from devastating chronic health impairment. Even decades after exposure, severe long-term effects like chronic obstructive lung disease, lung fibrosis, recurrent corneal ulcer disease, chronic conjunctivitis, abnormal pigmentation of the skin, and different forms of cancer have been diagnosed. **Methods:** This review briefly summarizes the scientific literature and own results concerning detection, organ toxicity of SM, its proposed toxicodynamic actions, and strategies for the development of improved medical therapy. **Results:** Despite extensive research efforts during the last century, efficient antidotes against SM have not yet been generated because its mechanism of action is not fully understood. However, deeper insights into these mechanisms gained in the last decade and promising developments of new drugs now offer new chances to minimize SM-induced organ damage and late effects. **Conclusion:** Polymerase inhibitors, anti-inflammatory drugs, antioxidants, matrix metalloproteinase inhibitors, and probably regulators of DNA damage repair are identified as promising approaches to improve treatment.

Sulfur mustard (SM; 2,2′-dichloroethyl sulfide; CASRN: 505-60-2) was first synthesized in 1822 by Despretz and modified in 1860 by Niemann and Guthrie.^1^,^2^ Only in later years, SM has been identified as a potent chemical warfare agent and was used at Ypres in 1915 during World War I for the first time. Synonyms are Hun Stoff distilled (HD), mustard gas (typical odor), Yperite (first use during the battle at Ypres), lost (acronym of the German chemists Lommel and Steinkopf, who investigated the mass production), Pyro (British code), and yellow cross (German shells were marked with a yellow cross).

Sulfur mustard is still the most abundantly produced and stockpiled vesicant worldwide. During the Iran-Iraq War (1983–1988), 100 000 Iranian soldiers were injured by SM attacks. At present, 10 000 of Iranians are now suffering from long-term adverse effects. Acute SM poisoning typically affects 3 major organ systems: skin, lungs, and eyes. Moreover, SM induced tissue damage of central nervous, gastrointestinal, and hematological system have also been reported.

The terrible experiences gained during World War I were the starting point of nearly a century of SM research. The research group of Goodman worked on the cytostatic and cytotoxic properties of SM and its nitrogen relatives during World War II.^3^ This research was declassified after World War II and resulted in medical applications of alkylating mustard compounds, for example, the first successful therapy of leukemia.^3^ The cytostatic effect of SM was also used to treat hyperproliferative skin diseases, for example, psoriasis.^4^ In addition, recognition of SM as an immunosuppressant compound prompted studies about chemical immunosuppression. These studies finally smoothed the way for organ transplantation.^5^

Nevertheless, despite some useful application in medical care the world is still facing the threat of military or, what seems to be even more likely, terrorist use of SM. Because the exact mechanism of SM pathology remains elusive, intensive research efforts have been made for 9 decades.

The aim of this article is to describe the clinical pathology and the proposed underlying pathophysiological mechanisms of SM toxicity. It focuses mainly on the acute epithelial lesions following SM exposure. On the basis of this concept, *rational targets* for further research are defined and options investigated by our group are shown.

## PHYSICOCHEMICAL PROPERTIES

Sulfur mustard is an oily liquid with poor solubility in water and a high solubility in organic solvents. Its color varies from light yellow to dark brown, depending on the technical impurities of the compound. Its freezing point lies between 13°C and 14°C and its boiling point between 215°C and 217°C (760 mm Hg). The physicochemical properties of SM are summarized in Table 1 Depending on technical impurities of the compound, a typical odor of the substance has been described as more mustard-, garlic-, or onion-like.

Sulfur mustard hydrolysis in water by a S_N_1 mechanism to form 2-hydroxyethyl 2-chloroethyl sulfide (HCES) and hydrochloric acid. HCES hydrolyses further forming thiodiglycol and hydrochloric acid. The rate constants in water at 25°C for the 2 consecutive hydrolysis reactions were estimated to be 2.933 ± 0.15 × 10^− 3^ and 3.87 + 0.14 × 10^− 3^ s^− 1^, respectively.^6^ In tissue, SM forms a cyclic sulfonium ion that alkylates nucleophilic cellular sites, leading to the pathology described later.

## TOXICOKINETICS

Penetration rates of liquid SM were determined in vitro (71–294 μg/(cm^2^/h)) on human skin) with a Franz-type glass diffusion cell that correspond quite well to in vivo data derived from human skin (60–240 μg/(cm^2^/h)).^7^ It could be shown that 80% of applied liquid SM evaporates before penetrating the skin.^8^ On the one hand, 20 μg/cm^2^ of liquid SM is needed to produce blisters,^9^ but only 4 μg/cm^2^ of vapor is sufficient to induce an equal effect.^10^ On the other hand, occluded conditions, sweat, and heat were shown to increase skin penetration dramatically.^7^ From the total penetrating SM only 10% to 20% are fixed to macromolecules in skin. The remaining 80% to 90% are rapidly transported away by circulation.^7^,^11^ Recently, evidence has accumulated that unhydrolyzed mustard even remains present in the stratum corneum and the upper epidermis.^7^ This finding could explain the occurrence of secondary blisters even 30 days after termination of SM exposure.^12^

Apart from severe local damages, SM may also cause systemic effects. A 2-compartment model was suggested for the elimination of SM from rats: *t*_1/2α_ = 5.56 minutes, *t*_1/2β_ = 3.59 hours, volume of distribution at steady state (*V*_dss_ 74.4 L.^13^ However, a high-distribution volume indicates accumulation of SM in fatty organs or fat depots. Redistribution of active SM may allow ongoing alkylation of various proteins, for example, hemoglobin. Indeed, it has been shown recently that the adduct levels of SM to hemoglobin in guinea pigs and marmosets increase over several days.^14^ A similar behavior may be observed in humans, as unhydrolyzed agent has been found in fatty tissues of an SM victim.^15^ In addition, blood samples taken 3 to 4 weeks after exposure showed substantial SM N7-guanine adducts in the DNA.^16^ This is rather surprising, as DNA adducts are more or less effectively repaired within 24 hours.^17^

In conclusion, these results provide strong evidences that unhydrolyzed SM may be slowly released from various tissue depots (eg, skin and fat). These data could provoke a paradigm shift as an SM reservoir in the body has not been assumed until now.^18^ The existence of an SM depot would have major implications for the treatment as well as for the safety of medical personnel treating casualties of sulfur mustard exposure. Therefore, the use of protective equipment is highly recommended, and rapid detection methods are required urgently. To fill this gap, a new SM detector based on an immunochromatographic test strip system was developed, as described later on in the course of this article.

## PATHOLOGY OF ACUTE SULFUR MUSTARD POISONING

Sulfur mustard pathology will be described in the following chapters deducing from gross to molecular pathology. Thereby, an integrated description of SM-induced effects will be attempted as summarized in Table 2.

## Gross pathology

The clinical effects of SM poisoning have been extensively reviewed.^19^–^22^ Acute toxic effects after SM exposure have been observed after a latency period of variable length depending on the dose, liquid, or volatile exposure, and the individual susceptibility. Three organ systems are known to be mainly affected: eyes, lung, and skin. Other early toxic effects have been described for the bone marrow, the central nervous system, and the gastrointestinal tract.

## Eyes

The irritating potential of SM on the eye was first discovered in 1887 by Theodor Leber (founder of ophthalmic research).^23^ The eyes are most sensitive to SM.^24^ The latency period is very short compared with all other organ systems. High concentrations may cause smarting of the eyes within 30 minutes.^19^ It is believed that reports on eye irritation appearing within minutes are due to impurities of SM. Mild ocular irritation can be observed after vapor exposure to doses of 12 to 70 mg·min/m^3^. Vapor concentrations of 50 to 100 mg·min/m^3^ cause ocular symptoms like conjunctivitis, grittiness under the eyelid, and tearing. As vapor doses increase (>200 mg·min/m^3^), eye injury is characterized by eyelid as well as corneal edema with impairment of vision, photophobia, and severe blepharospasm.^25^ However, the resulting temporary blindness of the patients will gradually improve after 4 to 5 weeks.^19^

## Respiratory tract

The respiratory tract is similarly sensitivity toward SM.^26^ Comparably low SM doses (12–70 mg·min/m^3^) may cause first symptoms. Where severity of signs and symptoms in skin and eye correlate with environmental concentration of SM, such a direct relationship does not hold true for the respiratory tract. Respiration rate affects the absorbed dose clearly. Inhalation of SM vapor mainly affects the laryngeal and tracheobronchial mucosa after a latency of several hours. The onset of symptoms starts with irritation of nasal mucosa, hoarseness, sneezing, and coughing. When fully developed, SM injury of the respiratory tract is characterized by lacrimation, rhinorrhea, loss of smell and taste, and discharge of mucus from nose and throat. As vapor doses increase, tracheobronchitis and pseudomembrane formation are observed. Pseudomembranes may loosen from the bronchiotracheal wall and cause obstruction of the airways or even provoke heart arrest (thus, leading to fatal outcome).^27^ As early as 1921, Koch described 1 case that showed a collapsed right lung as consequence of pseudomembranous obstruction of the right main stem bronchus.^28^ He reviewed the pathological findings in 62 cases of lethal gassing with SM. Eleven heavily SM-exposed soldiers died during the first 3 days of exposure. Eighty-two percent died within 2 weeks. In this report, fibrinous-hemorrhagic bronchopneumonias were described that tended to form abscesses. Sixty-six percent of all cases that survived the first 2 weeks showed lung abscesses. Even lung gangrene was observed in 3 of 62 cases. Koch proposed to divide the pathological sequence of SM-induced effects on the respiratory tract into 4 phases^28^:

1. catarrhal state;
2. pseudomembranous laryngotracheitis state;
3. pseudomembranous bronchitis and bronchopneumonia state; and
4. lung abscess and gangrene state.

At highest vapor concentrations also symptoms of the lower respiratory tract infections, like pulmonary edema, were described,^25^,^26^ which might even progress into the full picture of adult respiratory distress syndrome.^27^ Pulmonary emphysema has been frequently observed and has been reported to be of alveolar, interstitial, and mediastinal nature.

## Skin

The skin's susceptibility to SM exposure mainly depends on three factors: skin temperature, moistness, and anatomical location. Thus, moist body areas with a thin epidermal layer (eg, scrotum, anal region) appeared to be highly sensitive to SM vapor. The onset of symptoms depends on the absorbed agent dose. Higher doses are known to shorten the symptom-free latency period. Erythema can frequently be observed 4–8 hour after SM exposure at a threshold dose (vapor: 100–300 mg·min/m^3^, liquid: 10–20 μg/cm^2^) while blister formation occurs at higher doses (vapor: 1000–2000 mg·min/m^3^, liquid: 40–100 μg/cm^2^).^29^ Blisters appear as small vesicles in the erythema area and coalesce to large bullae. The blisters are typically thin-walled and filled with a clear yellow fluid. The affected skin areas show a positive Nikolsky sign, which means that increased physical friction aggravates local damage. It is reported that skin blistering may last for several weeks after exposure despite any further contact to SM.^12^

Acute cutaneous SM lesions have been classified as follows^19^:

1. erythematous form,
2. pigmentary exfoliation,
3. superficial vesicular to bullous form,
4. bullous necrotization,
5. deep necrotizing nonbullous form, and
6. allergic and toxic contact reactions of the skin.

Pigmentation disorders are frequently observed. Hyperpigmentation can persist in the affected area for decades. However, hypopigmentation as well has been commonly observed and may be located next to hyperpigmented areas. The resulting landscape-like appearance (poikiloderma) of the skin is a characteristic dermal late effect after dermal SM injury.

## Other organ systems

The symptoms of systemic poisoning are very similar to those caused by radio- or chemotherapy. SM may induce headache, nausea, vomiting, and loss of appetite. The gastrointestinal tract and the bone marrow are heavily damaged at higher doses. Thus, immune suppression, leukopenia, diarrhea, fever, cachexia, and, in very severe, cases excitation of the central nervous system with convulsions have been described.^19^,^25^

Taken together, in all affected organ systems, inflammation and tissue destruction are the most prominent pathological phenomena.

## Histopathology and cytopathology

SM mainly enters the body via several epithelial tissues: the skin, the eye (corneal and conjunctival), and the respiratory tract. These epithelial cells are exposed toward highest SM concentrations. Nevertheless, cells of the underlying tissue (eg, endothelial cells) are also affected.

### Symptoms

The ocular epithelium does not form a barrier like the stratum corneum in the skin. Thus, SM penetrates more easily through the ocular epithelia. Several hours after SM exposure, conjunctival and corneal edema has been frequently observed. Goblet cells disappear leading to decreased production of conjunctival mucus. Conjunctival vessels are occluded as a result of severe endothelial damage. The corneal epithelium begins to detach from its stroma and small vesicles are formed. Corneal-free nerve endings are directly affected resulting in severe ocular pain and blepharospasm. At more severe exposure, extended destruction of the limbal blood vessels has been observed^24^ and chemical anterior uveitis, corneal necrosis, and lens opacification were reported.^30^

### Respiratory tract lesions

Only scarce data are available concerning the effects of SM on the human respiratory tract at microscopic level. Data are available only from patients who died several days or weeks after exposure. Thus, primary damage and secondary effects are difficult to distinguish. The pathological findings in casualties from lethal gassing during World War I and the Iraq-Iran War showed similar findings.^31^,^32^ One of the most complete descriptions of the pathology of human respiratory tract lesions after SM exposure is provided in the study by Koch, which was previously noted.^28^ SM affected more the upper part of the respiratory tract and only severe exposed persons showed signs of deep pulmonary damage. The catarrhal state was not observed in pure form as most cases already showed formation of pseudomembranous laryngotracheitis. Pseudomembranes were composed of fibrin and cell debris derived from infiltrating leukocytes and necrotic epithelium. Koch concluded that this state may probably only be observed during day 1 after SM exposure. The pseudomembranous laryngotracheitis was characterized by diphtheria-like inflammation with fibrinous deposits. Mucus was observed in the upper respiratory tract: nose, throat, larynx, glottis, and upper parts of the trachea. Paranasal sinuses were affected with varying degree. The epithelial lining of the upper respiratory tract showed signs of necrosis. During day 3–6, necrotic cells appeared in the whole upper respiratory tract. A thick continuous membranous layer was observed lining the uvula, tonsils, epiglottis, pharynx, larynx, and bronchi. Massive leukocyte infiltration was described leading to bronchial obstruction.^33^ The lungs mostly showed no signs and symptoms. A prominent feature was the engorgement of the blood vessels. The alveoli exhibited signs of emphysema. Only severe SM intoxicated patients showed signs of lung edema. Available animal data are in line with the described pathological sequence in man.^33^,^34^

In general, SM-induced damage of the respiratory tract is also characterized by edema, inflammation, and cell death of the airway epithelial lining. The main difference to the observed skin effects is that lung pathology is characterized by great mucus production.

### Skin lesions

The skin is composed of three primary layers: epidermis, dermis, and hypodermis. SM affects mainly the outermost layer (epidermis) as it could be detected at the microscopic level. Cytotoxic effects have been firstly noted in the highly proliferative basal keratinocyte layer.^25^,^33^,^35^ Seperation of epidermis and dermis has been observed after several hours. In this state the stratum corneum was described to be edematous and the basal layer seemed to be intact without pathological findings except for some irregular nuclei.^25^ The nuclear morphology of the basal layer was characterized by karyolysis and pyknosis. Nuclear karyorrhexis was less noted.^36^ The dermis was less affected and showed only signs of discrete necrosis, together with a decreased number of fibroblasts and histiocytes. Biopsies taken from an erosive zone exhibited no epidermis. Necrosis and massive cellular infiltration were described. Interestingly, capillary engorgement as well as thrombosis could be seen.^25^,^36^

SM-induced damage to the skin is also characterized by edema, inflammation, and cell death mainly of the basal keratinocyte layer. The main difference to the observed pulmonary effects is that the skin pathology shows less infiltration with leukocytes.

Taken together, in all affected organ systems three histopathological observations could be made: cell death, seperation of cellular layers, and cellular infiltrate.

## Molecular pathology

Although during one century of medical research several hypotheses of SM-induced pathological effects were produced, none of these hypotheses have been completely accepted. It is likely that various proposed mechanisms identified so far may be active at the same time and be part of a complex picture, which is not entirely understood at present.

## Cell death

The so-called acid liberation theory was one of the first hypotheses on SM-related cell injury. According to this theory, both 2-chloroethyl-side chains of the SM molecule undergo first order (S_N_1) intramolecular cyclisation resulting in formation of hydrochloric acid in an aqueous environment. The proposed intracellular acidification was held responsible for the subsequently observed cellular damage.^37^ However, as vesicant action does not correspond to the rate of acid liberation, it has been assumed that acid formation does not play a major role.^38^ Nonetheless, the acid liberation theory has recently gained new interest. Sawyer et al showed that keratinocytes better survived SM exposure at basic pH-values.^39^ The protective effects of alkalization observed in vitro are outstanding and need further investigation. Apart from acid formation, the ethylene sulfonium cation intermediate has been assumed to open to form a highly reactive carbenium ion, which immediately reacts with different cell constituents like DNA, RNA, proteins, and other molecules. It has been proposed that especially the reactions of SM with proteins and inhibition of several enzymes significantly contribute to the SM-induced cytotoxicity.^40^,^41^ Extensive studies revealed that hexokinase was one of the most susceptible enzymes. The finding that other vesicant substances inhibit hexokinase substantiated the SM data. Investigations of the SM effects on this enzyme were performed with a purified crystalline hexokinase isomers (I–III). Hexokinase IV (50 kD) has not been purified so far. Dixon and Needham showed that 6–7 alkylations within every hexokinase molecule were sufficient to completely block enzyme activity.^42^ However, it was calculated that the SM concentration needed for complete enzyme inhibition in vitro does not correlate with the vesicant doses in vivo. Similar to the “acid hypothesis,” hexokinase inhibition furthermore should result in immediate cellular damage due to energy shortage.^43^

SM is effectively eliminated by glutathione. Thus, high SM concentrations can rapidly deplete the intracellular glutathione levels, resulting in the enhanced production of reactive oxygen species.^44^ Consequently, pretreatment of cells with various antioxidants does not only enhance cell survival^44^,^45^ but antioxidants have also been shown to be most effective in treating SM lung injury in animal models.^46^–^48^

The nucleus is regarded as the most SM-sensitive cell component.^49^ Several reactions affect the DNA by forming mono- and bifunctional SM adducts. Sixty-one percent of all alkylations refer to N7 of guanine forming 7-(2-hydroxyethylthioethyl) guanine (7-HETE-G).^50^–^52^ Niu et al demonstrated that at a SM concentration of 2.3 μM one 7-HETE-G molecule per 1 million nucleotides is produced.^53^ In addition, SM also alkylates position 3 of adenine (16%) and O 6 of guanine (0.1%).^54^ Apparently, human DNA repair mechanisms are not able to remove O6-(2-ethylthioethyl) guanine. Thus, this mechanism has been accounted for the mutagenic effects of SM. Nearly 17% of the total of alkylations produce intra- or interstrand cross-links.^55^ These multiple alkylations cause phosphorylation of ataxia teleangiectasia mutated protein at serine 1981. In consequence the histone H2Ax is phosphorylated and p53 accumulates in the cell.^56^ The degree of SM-induced DNA damage is decisive for the further cell fate. With increasing SM concentration cellular responses consists of cell cycle arrest, terminal differentiation, apoptosis, or necrosis.^57^ SM-injured cells are arrested at certain cell cycle checkpoints. At higher concentrations (>50 μM), G1 block predominates whereas G2 block occurs at 10-fold lower SM concentrations.^58^ Genotoxic stress induced by SM stimulates DNA repair.^59^ Failure of DNA repair might result in programmed cell death either by terminal differentiation or via apoptosis.^60^–^64^

In any case of DNA damage, an early burst of (ADP-ribose) polymer formation by activation of poly(ADP-ribose) polymerase type 1 (PARP-1) can be observed that consumes its substrate nicotine adenine dinucleotide (NAD^+^), which is resynthesized by ATP.

At moderate DNA damage, the cell is able to counteract the increased PARP-1 activity by its own means. PARP-1 is a caspase 3 substrate in the early phase of apoptosis.^65^ Cleavage of PARP-1 eliminates its enzyme activity and preserves the cellular energy pool.^66^

At high SM concentrations, PARP-1 is excessively activated resulting in rapid NAD^+^ and ATP depletion,^67^,^68^ which is associated with necrotic cell death.^69^–^72^

A pharmacological approach to reduce PARP-induced necrotic cell death is the use of PARP inhibitors. PARP inhibition also blocks DNA repair^59^ and thereby promoting cell death by apoptosis.^73^

Furthermore, Ca^++^ chelators can attenuate the apoptotic response.^57^ Bellomo et al proposed that modification of protein thiols might be an important event for the inhibition of microsomal Ca^++^ sequestration caused by a variety of toxic agents.^71^ Orrenius recently reviewed the impact of Ca^++^ changes on cell death.^74^ Evidence accumulated that oxidation of protein thiol groups from mitochondria may open a voltage-dependent anion channel (VDAC). This event releases not only Ca^++^ but also low-molecular-mass matrix components. The same mechanism has also been shown for SM-mediated cytotoxicity.^75^

## Separation of cellular layers

The activation of several proteases and proinflammatory cytokines is a further important mechanism of SM injury.^76^–^78^ The formation of large blisters after SM injury shares some similarities with epidermolysis bullosa.^79^ Former studies revealed that 24 hour after SM exposure, a discontinuous pattern of laminin 5 and type VII collagen could be observed.^80^ Interestingly, some forms of junctional epidermolysis bullosa (JEB) have been connected to laminin 5 mutations. Furthermore, ultrastructural analysis revealed that in both pathologic sequalae the epidermal-dermal junction separation occurs within the lamina lucida.^79^ Hemidesmosomes contain two proteins that can be used to characterize the blister plane. BP230 (also known as BPAG1) is an intracellular protein that promotes the association of hemidesmosomes with keratin intermediate filaments. BPA immunoreactivity is diminished in SM-exposed guinea pig skin.^81^ BP180 is a transmembrane protein with a collagenous carboxyl-terminal extracellular domain.^82^ Immunoelectron microscopic studies demonstrated that the C-terminal part of BP180 actually reaches into the lamina densa.^83^ Immunhistochemical studies revealed that BP180 was present in both the epidermis (blister roof) and dermis (blister ground) of mice skin following SM exposure.^80^ This supports in part the findings of earlier studies that described intact hemidesmosomal components and attached anchoring filaments to be forming the roof of the blister and the lamina densa its base.^76^

Recently, it has been shown that, for example, matrix-metalloproteinases are main players in skin pathology of SM-induced blisters^84^,^85^ and in JEB.^86^ Studies with the mouse ear model furthermore suggest that matrix metalloproteinase-9 (MMP-9) is the most upregulated MMP in SM exposed skin.^84^ Neutrophil-derived MMP-9 inactivates α 1-proteinase inhibitor, which is known to be the endogenous inactivator of neutrophil elastase (serine protease). MMP-9 and elastase are both capable of cleaving BP180. Recent studies have demonstrated that the effect of MMP-9 appears to be more indirect one, lying upstream of the neutrophil elastase.^87^

Interestingly, MMP activation and massive infiltration with neutrophils has also been found in SM-damaged lungs.^88^,^89^ Especially, gelatinase B activity (MMP-9) was elevated 24 hours after SM exposure. Meanwhile, treatment with the MMP-inhibitor doxycycline attenuated lung injury.^88^ In the eye, MMP activation after SM exposure could also be shown and pathologic effects could be ameliorated with the MMP inhibitor Ilomastat.^90^

## Inflammatory mediators and cellular infiltrate

The histopathology of SM-damaged organs indicates that various vasoactive and chemoattractant mediators are produced in the affected area. Several pathways are discussed to regulate gene expression of proinflammatory mediators. Dillman et al have demonstrated that p38 MAP kinase signaling is involved in proinflammatory cytokine release.^91^ IL-1α/β, IL-6, IL-8, TNF-α, and GM-CSF have been shown to be released shortly after SM exposure.^85^,^92^–^95^ This cytokine release pattern is known to have a strong chemotactic activity for neutrophils and macrophages. The amount of infiltrating cells is, therefore, both dose and time dependent.^80^ Nowadays, anti-cytokine drugs (eg, infliximab, etanercept) are available and should be tested in currently available in vivo animal models of SM injury.

Another group of inflammatory mediators are the eicosanoids, which are generated de novo from phospholipids. The most important eicosanoids are the prostglandins, thromboxanes, and leukotrienes, which are all involved in SM-related tissue damage. Cyclooxygenase (COX-1, COX-2) inhibition has been shown to reduce SM-induced inflammation.^96^ As the anti-inflammatory action of glucocorticoids largely results from inhibition of cyclooxygenase inhibition, glucorcoticoids also provided some protection.^97^,^98^ However, detailed studies are still needed to evaluate the best therapeutic regimen to ameliorate or reduce SM-induced inflammation.

## STRATEGIES FOR THE DEVELOPMENT OF IMPROVED DIAGNOSIS AND THERAPY

From our point of view, it appears rational to investigate

1. decontamination,
2. diagnosis,
3. pathophysiology,
4. effect monitoring,
5. development of new toxicological methods, and
6. therapeutic approaches.

### Decontamination

The best strategy to protect a person against the various toxic effects of SM is to prevent or minimize contamination with the agent. Protective gears, mashes, and special rubber boots and gloves provide good protection against SM vapors for a certain period. Several countries have developed either topical skin protectants (TSP) or decontamination devices. TSPs are applied to skin prior to exposure to minimize penetration of CWAs. Some formulations contain chemicals, which degrade CWAs into nontoxic compounds. However, the use of such TSPs is only an option for persons who are priory aware to enter a contaminated environment, for example, soldiers, fire workers, medical personnel, and others. People attacked by surprise have to rely on effective decontamination procedures. Studies from World War I had shown that the application of a solvent could prevent vesication up to 45 minutes postexposure.^99^ In contrast, in studies performed during World War II it was found that SM was rapidly fixed to tissue structures. Peeling off the intoxicated skin has been shown to minimize vesication even 14 hours postexposure.^100^ This implicates a significant persistent reservoir of reactive SM in exposed skin. More recently, Chilcott has shown that 35% of the applied SM dose is present in the upper epidermis and stratum corneum.^101^ This SM reservoir has to be extracted and is not affected by decontamination procedures using tap water, reactive powders, neutralizing solutions, reactive skin lotions (eg, RSDL), or absorbent powders (eg, fuller's earth). The above-described findings have major implications for medical procedures and safety measures of medical personnel. Several research projects are dedicated to improve decontamination of CWA. It is believed that the novel use of enzymes provides important advantages in a medical context compared to already-existing harsh chemical procedures. Recently, Amitai et al showed the efficacy of a new enzymatic system, using chloroperoxidase to degrade SM and VX.^102^

### Detection of exposure

The ability to detect traces of chemical warfare agents has become a greater priority in recent technological and biotechnological research. A reliable diagnostic tool to monitor and confirm the presence of SM even after decontamination procedures is still being needed. In this context, continuous air monitors using gas chromatography (Minicams™) are used but have been found to lack sensitivity. In addition, these detectors are expensive and need special training. By contrast, immunochromatographic test strip systems have shown several advantages over other detection methods, most notably the easy on-site use, the small size of the detector, and the rapid test results. A feasibility study to develop a small prototype detector has been initiated in cooperation between the Securetec AG, Munich, and the German Armed Forces Medical Corps. The aim of this project is the development of such an immunochromatographic test strip for the reliable and rapid detection of sulfur mustard under operational conditions, including contaminated areas, for example, following terrorist attacks.

In an aqueous environment, SM quickly reacts with DNA and forms in 61% alkylations to N7 of guanine forming 7-(2-hydroxyethylthioethyl) guanine (7-HETE-G).^50^–^52^ In our immunochromatographic detection system, a well-characterized antibody (2F8) was used, which demonstrated high specificity and sensitivity for SM ssDNA adducts in ELISA assays.^14^ In the first project phase the antibody was purified and conjugated to colloidal gold particles that are used for visualization of the test line. Preliminary experiments showed that 2F8 conjugates bind specifically to SM-treated ssDNA and run well in the lateral flow technology system.

**Figure 1:**
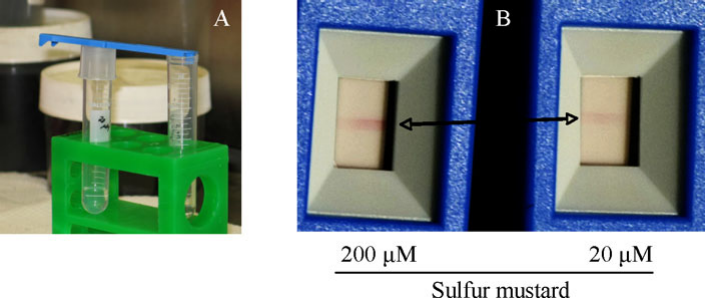
Detection of sulfur mustard vapor with the sulfur mustard detector. Sulfur mustard was diluted in phosphate-buffered saline at indicated concentrations. Sulfur mustard detector was held above the fluid for 30 seconds (A). The sulfur mustard detector showed a red line as a positive result (B).

**Figure 2:**
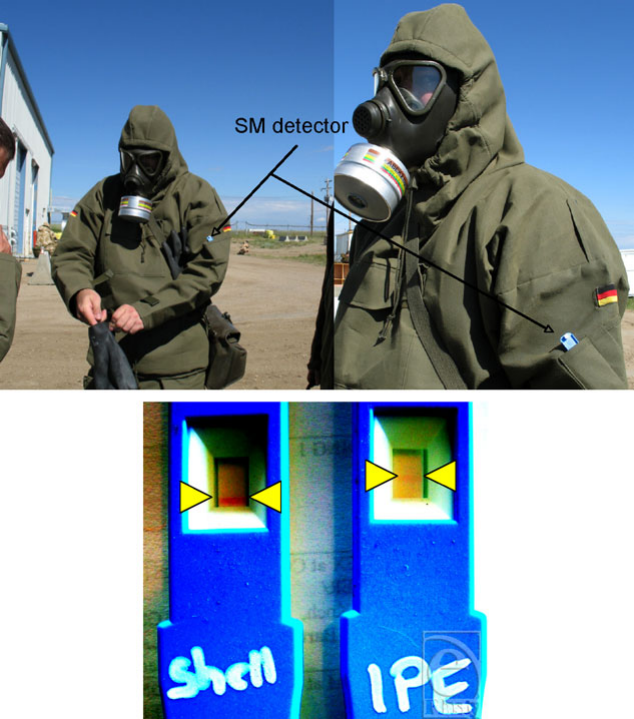
Sulfur mustard detector was used to detect environmental traces of sulfur mustard. Sulfur mustard detector was attached to an individual protective equipment before the soldier entered a cave with an open sulfur mustard grenade shell. Sulfur mustard detector was held over the shell for 5 seconds. After leaving the cave, the sulfur mustard detector showed red lines as positive results for the individual protective equipment as well as for the shell.

Free SM quickly reacts with oligonucleotides attached on the strips forming adducts predominantly at the N7-guanine. These adducts were visualized using the lateral flow technique to form a clearly visible line.^103^ The SMD was able to detect SM vapor released from a 20 μM solution (Fig 1) and from pig skin exposed to 2 μM SM diluted in phospate buffered saline. It was sufficient to hold the SMD near the skin surface (data not shown). As the SMD detects free SM, we considered the possibility of using it in a contaminated area. For this purpose, the SMD has been tested during a NATO CBRN Defence Live-Agent exercise. The SMD was attached to the individual protective equipment of a soldier who entered a cave. The soldier found an open shell with an unknown liquid and used the SMD to confirm the presence of SM. The SMD showed positive results for the shell as well as for the detector attached to the IPE (Fig 2).

The prototype SMD has been shown to detect SM on skin and in the environment. This easy-to-use detection system will be improved in an additional study.

### Pathophysiology

Although a huge quantity of data exists, SM pathophysiology is still not clearly understood and further research has to be done. Presently, our group investigates the relevance of mode of cell death, MMPs, and inflammatory response on SM-induced cell injury.

### Mode of cell death

To evaluate apoptosis and necrosis, ROS, NO, and further signal molecules in SM-treated cells, a screening program has been set up. In this program, Bloch et al were able to show the activation of the NO-producing enzymes eNOS and iNOS, as well as the formation of free radicals and radical reaction products. Furthermore, the detected activation of caspase-3 and the 85-kDa cleavage product of PARP indicated the induction of apoptosis.^104^

### MMPs

Popp et al focused on the expression and activity of the two gelatinases MMP-2 and MMP-9, which preferentially cleave collagen type IV. This major component of basement membranes is present at the interphase of the epidermis and dermis which are both affected upon SM exposure. In this study, mRNA expression levels (using real-time quantitative PCR) and protein synthesis (by means of zymography and Western blotting) of both MMP-2, MMP-9, membrane-type 1 MMP (MT1-MMP), and the major physiological MMP inhibitors TIMP-1 and TIMP-2 were determined in various human cell lines such as keratinocytes (HaCaT) and fibroblasts. The results revealed different patterns of expression for MMPs and TIMPs in the investigated cell lines compared with primary cells indicating for a distinct regulation of the corresponding genes upon treatment with 1–100 μM SM.^105^

### Inflammatory response

Rebholz et al showed that SM activates NFkB in a biphasic manner in mouse keratinocytes. This effect was preceded by phosphorylation of IKKβ, Iκ Bo, and RelA. Further downstream, it could be shown that gene expression of IL-1β, TNF, and other NFkB dependent factors is activated.^106^

### Effect monitoring

SM induces alkylation of the DNA, thus producing DNA mono- and diadducts (ie, DNA strand breaks and interstrand cross-links). These DNA modifications are believed to be highly mutagenic and carcinogenic. Quantification of DNA strand breaks and DNA cross-links in living cells is, therefore, an important endpoint in the assessment of SM toxicity. Appropriate monitoring, however, has so far only been restricted to rather time-consuming and difficult methods such as the comet assay.^107^ However, Debiak et al just recently adapted a robust, fluorescent-based method for the detection of DNA damage also apt for high throughput applications.^108^ This fully automated version of the Fluorescent Alkaline DNA Unwinding (FADU) assay is faster and more sensitive than currently used methods. The assay is running on the base of a 96-well format, allowing parallel analysis of multiple samples. The procedure is completed within 2–4 hours including sample preparation. The high sensitivity, high throughput, fast and easy handling, and low costs make the FADU assay an attractive candidate for the assessment of SM exposure of victims.

### Development of new toxicological methods

SM injury is a rather rare intoxication, with only a few people having been affected worldwide. Hence, reliable human data are scarce, and SM exposure of animals is hampered by long observation times. To reduce or replace animal experiments for elucidation of SM-induced pathomechanisms and relevant therapeutic approaches, several in vitro models were developed to study especially SM airway injury. In this context, just recently a coculture model of the human distal lung consisting of human epithelial and microvascular endothelial cells has been established to study cellular interactions of the epithelium and endothelium at the alveolo-capillary barrier.^109^ This in vitro model has been shown to be a suitable means to examine epithelial and endothelial interactions in the pathogenesis of acute lung SM injury. A concentration-dependent increase of SM-mediated cytotoxic effects with high affection of endothelial cells could be demonstrated.^45^

### Therapeutic approaches—the road ahead

Substantial progress has been made to improve medical treatment of SM skin injury in the past.^22^ However, the most serious long-term effects of SM intoxication are respiratory disorders. The respiratory tract is more difficult to treat in comparison to skin burns. Thus, it is necessary to enhance research efforts to identify pharmacological targets with more relevance to lung injury. Inflammation and cell death are prominent features of SM injury. It has been shown that steroids and nonsteroidal anti-inflammatory drugs are beneficial.^22^ Therefore, it is crucial to get a better insight in activation of NFkB and release of prostaglandins. In addition, MMP activation is present in all SM-affected organ systems. Detailed insight concerning the regulation of MMP activation and other proteases is still needed. Nevertheless, potent MMP inhibitors are available and are of possible relevance to prevent SM lung damage.^88^ Besides MMP inhibitors, *N*-acetylcysteine is a potent drug to treat pulmonary lesions after SM inhalation. Thus, more information is needed concerning the relevance of reactive oxygen species and nitric oxide formation. There is no doubt about a beneficial effect of slowing down inflammation and tissue destruction by MMPs after SM exposure. In contrast, it is difficult to judge upon the final outcome of pharmacologic treatment of SM induced cell death and DNA damage. It has been successfully shown to inhibit SM-induced apoptosis in vitro by caspase inhibition.^110^ The rationale of this intervention is to give the cell time for DNA damage repair. It has to be shown that initiation of apoptosis is associated with a loss DNA repair abilities, as related regulatory proteins; for example, PARP are cleaved in this phase. If apoptosis is inhibited, it can be assumed that cells with a damaged or broken DNA will either die a necrotic cell death, due to persistent DNA damage and ATP depletion, or survive. In case of necrosis, this would enhance the inflammatory response dramatically and promote further tissue damage. In case of cell survival, cells may have irreversible levels of DNA damage, which are later prone to mutagenic transformation. Thus, it would be perhaps better to eliminate SM-damaged cells as soon as possible, which in case of SM skin injury is achieved by surgical wound debridement, which also promotes wound healing.^22^ However, this surgical procedure will not be available for mass casualties. In addition, lungs and eyes cannot be treated in this way. Thus, a pharmacological approach is still needed. Summarizing, the drug or drug combination should diminish the inflammatory response, prevent survival of DNA-damaged cells, minimize tissue destruction, and enhance wound healing.

An interesting drug family, which should be considered again in this context, is pharmacological inhibitors of PARP, which have the potential to promote apoptosis, reduce cell necrosis,^63^,^111^ and downregulate multiple simultaneous pathways of inflammation and tissue injury.^112^ By suppressing inflammatory response and inhibiting infiltration of activated mononuclear cells, PARP inhibitors could indirectly diminish oxidative and nitrosative stress.

**Figure 3:**
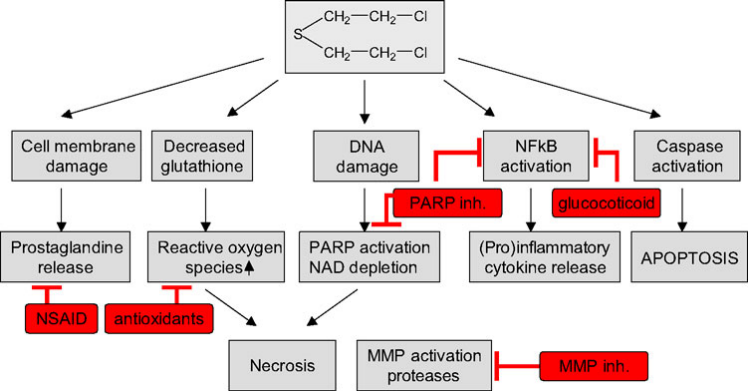
Pathways implicated in sulfur mustard-induced pathophysiology and possible targets (red) for therapeutic intervention. Sulfur mustard-induced direct and indirect (reactive oxygen species) DNA damage lead to polymerase (PARP) activation and nicotine adenine dinucleotide (NAD) depletion, which may result in necrotic cell death. Sulfur mustard exposure has also been demonstrated to activate the extrinsic and intrinsic pathway of apoptosis. Sulfur mustard-induced release of (pro)inflammatory cytokines has been linked to NFkB activation and prostaglandine release. Sulfur mustard has also been shown to upregulate matrix metalloproteases and serin proteases. The exact signal transduction for matrix metalloproteinase (MMP) activation has not been identified yet. In conclusion, PARP inhibitors, anti-inflammatory drugs, antioxidants, and MMP inhibitors are identified as promising pharmacological approaches to improve clinical outcome.

In summary, with respect to present knowledge about SM-induced pathophysiology PARP inhibitors, anti-inflammatory drugs, anti-oxidants, MMP inhibitors, and probably regulators of DNA damage repair are identified as promising approaches to improve treatment (Fig 3). Safety considerations concerning long-term effects of drug treatment, which might affect DNA repair, mutations, and even epigenetic mutations, have to be taken into account. Moreover, it is a long way to introduce such drugs into medical treatment. Clinical studies and bridging studies are needed. This has not been done or initiated so far. To achieve this goal, a coordinated multinational approach would useful to compile dossiers for drug regulatory purposes, sharing the financial burden and avoiding duplication of effort.

## Figures and Tables

**Table 1:** Physicochemical properties of sulfur mustard

Chemical formula	C4H8Cl2S
Appearance	Oily liquid, light yellow to dark brown
Odor	Mustard, garlic, and onion
Molecular weight	159.08
Liquid density	1.27 (specific gravity)
Freezing point	13°C–14°C
Boiling point	215°C–217°C
Volatility (mg/m^3^, 20°C)	610
Solubility	Poor in water, high in ethanol

**Table 2:** Synopsis of sulfur mustard pathology

Gross pathology	Histopathology and cytopathology	Molecular pathology
Erythema	Cellular infiltrate	cytokines (IL-1, IL-6, IL-8, and TNF-α)
Pain	Separation of cellular layers	Prostaglandins
Blisters	Apoptosis	Matrix metalloproteinases
Pseudomembranes	Necrosis	Serine proteases
Ulcers		Caspase activation
Impaired wound healing		DNA adducts
		Cell cycle arrest
		Oxidative stress
		Intracellular Ca^++^ increase
		Impaired energy metabolism
